# A multi-element flame retardant containing boron and double-bond structure for enhancing mechanical properties and flame retardancy of epoxy resins

**DOI:** 10.1038/s41598-024-58709-0

**Published:** 2024-04-07

**Authors:** Penglun Zheng, Haihan Zhao, Junwei Li, Quanyi Liu, Jian Zhang, Wencai Wu

**Affiliations:** 1 College of Civil Aviation Safety Engineering, Civil Aviation Flight University of China, Guanghan, 618307 People’s Republic of China; 2 Civil Aircraft Fire Science and Safety Engineering Key Laboratory of Sichuan Province, Guanghan, 618307 People’s Republic of China; 3 Sichuan Key Technology Engineering Research Center for All-Electric Navigable Aircraft, Guanghan, 618307 Sichuan China; 4 China Academy of Civil Aviation Science and Technology, Beijing, 100028 People’s Republic of China

**Keywords:** Epoxy resin, Multi-element flame retardant, Double-bond, Crosslinking, Chemical safety, Materials chemistry

## Abstract

A multi-element synergistic flame retardant with double-bond structure was synthesized and added to epoxy resin (EP) to obtain EP composites with high flame retardant and mechanical properties. The study demonstrated that the DOPO-KhCPA-5 composite, containing 5 wt% of DOPO, exhibits the limiting oxygen index (LOI) value of 32%, indicating a high resistance to combustion. Additionally, it successfully meets the UL-94 V-0 grade, indicating excellent self-extinguishing properties. The DOPO-KhCPA-5 compound exhibited a 48.7% decrease in peak heat release rate (PHRR) and a 7.2% decrease in total heat release (THR) compared to pure EP. The inclusion of double-bonded architectures in the DOPO-KhCPA-5 composites led to a significant enhancement in both the tensile strength and tensile modulus. Specifically, the tensile strength increased by 38.5% and the tensile modulus by 57.9% compared to pure EP. This improvement can be attributed to the formation of a fully interpenetrating network of macromolecular chain structures by DOPO-KhCPA within the EP matrix. This network increased the entanglement between molecular chains, resulting in positive effects on the mechanical properties of the EP. Multi-element of DOPO-KhCPA exhibits a synergistic effect, providing condensed and noncombustible gas-phase flame retardancy. Additionally, the mechanical properties were improved with the introduction of flame retardants due to the good impact of double-bond cross-linking. The effectiveness of DOPO-KhCPA as an additive for developing high-performance EP with significant potential applications has been proven.

## Introduction

Polymer materials are closely related to our daily life, due to the excellent physical, mechanical and electrical insulating properties, and adhesion to other materials^1–3^, which some materials do not have. Among them, epoxy resins (EPs) are widely used in various fields such as coatings, adhesives, and molded composites due to their excellent properties and already mature manufacturing processes^4,5^. Nevertheless, pure EP is an extremely combustible substance that emits a significant quantity of smoke and harmful fumes when ignited. Hence, the examination of fire safety holds significant significance. Initially, halogen-containing flame retardants were employed to enhance the flame retardant capabilities of polymer materials. However, this practice results in the emission of a substantial quantity of toxic gases and substances during the combustion of the materials, leading to environmental pollution and degradation^6–8^. Therefore, flame retardants are developing in the direction of halogen-free, low-toxicity, environmental protection and high efficiency.

Halogen-free flame retardants typically include phosphorus, nitrogen, silicon, boron and metal flame retardants. Phosphorus flame retardants primarily function as flame suppressants by generating free PO radicals, which halt the combustion chain reaction in the gas phase^9–11^. They also interact with degraded polymers to enhance the carbon yield of the condensed phase. The nitrogen flame retardant can undergo thermal decomposition to produce non-combustible gas, which acts as a heat-absorbing and oxygen-insulating agent, so achieving the objective of flame retardancy^12–14^. When exposed to heat, silicon flame retardant forms a dense and stable silicon-contained carbon layer. The layer acts as a heat insulator and oxygen barrier, preventing the thermal degradation of polymer materials. Boron flame retardants are mainly used to seal the surface of flammable materials through the formation of a carbon layer to achieve a flame retardant effect^15–18^. Furthermore, it can alter how flammable materials break down under heat and prevent the formation of flammable gases, thus achieving the objective of flame retardancy. These halogen-free elements can successfully function as flame retardants. However, it is becoming increasingly difficult for flame retardants containing only one flame retardant element to meet actual production requirements. Research has discovered that the combined effect of various flame retardant elements can better achieve flame retardancy and also improve the mechanical properties of the EP composites^19–23^.

Designing a flame-retardant structure containing cross-linkable groups can also achieve the effect of enhancing the flame-retardant effect without affecting the mechanical properties. In our previous study, the theory of enhancing flame retardancy and compatibility by cross-linking the flame retardant itself was proposed, and the effect of cross-linking flame retardancy was confirmed by designing flame retardants containing cross-linkable phthalonitrile groups. It has been shown through further research that the process of double-bonded cross-linking at low temperatures can create a complex chain structure within the material and enhance the interlocking of chain segments^24,25^. It significantly mitigates the adverse effects of the flame retardants added to the EP. Specifically, a flame retardant called DCSA-Cu, which contains the double-bond structure and organometallic metal was developed^26^. The results proved that the flame retardant containing double-bond structure can promote the formation of macromolecular chain structure within the material, and effectively reduce the adverse impact of flame retardants on the mechanical properties of EP while obtaining a good flame retardant effect. This illustrates that the incorporation of a double-bond structure in a flame retardant containing multiple elements can further reduce the polarity of the flame retardant.

Therefore, the multi-element synergistic flame retardant was prepared using cinnamaldehyde, γ-Aminopropyl triethoxysilane (KH550), 9,10-Dihydro-9-oxa-10-phosphaphenanthrene 10-Oxide (DOPO) and phenylboronic acid (PA) as the raw materials for the reaction. P/N/Si flame retardants with the double-bond structure was synthesized by using the reactive P–H group on DOPO and the "aldol-ammonia condensation" reaction. When added to EP, the double-bond can form a large molecular chain structure through cross-linking, forming a dense network structure in the EP matrix and increasing the entanglement. And the addition of phenylboronic acid makes the flexible Si–O–B structure in the flame retardant. Finally, a new multielement synergistic flame retardant with excellent flame retardant properties and toughening effect was obtained and introduced into EP to study the effect on the properties of EP composites.

## Experiments

### Materials

Diglycidyl ether of biphenol A (DGEBA, E51) was buy from Shandong Jia Ying Chemical Co., Ltd., China. KH550, Cinnamaldehyde, DOPO, PA, 1,2-Dichloroethane, Ethanol, formic acid, 4,4′-diaminodiphenylmethane (DDM) were manufactured by Shanghai Aladdin Biochemical Technology Co.,Ltd, and none of them were subjected to secondary treatment.

### Preparation of DOPO-KhCPA

A three-necked flask was placed in an oil bath, nitrogen was introduced, KH550, cinnamaldehyde, DOPO were added in substance amount of 1:1:1, and solvent ethanol was added (0.05 mol DOPO corresponded to the addition of 150 ml of ethanol) while the oil bath was warmed up to 70 ℃ with stirring. When the solids are dissolved, increase the temperature of the frying pan to 90 °C, add the appropriate amount of formic acid (50 ml ethanol with 2 ml of formic acid), at the reaction time of 4 h, filtration precipitation with ethanol washing was performed and vacuum drying was carried out within 100 °C to obtain the precursor phosphorus-nitrogen-silicon synergistic flame retardant.

**Figure 1:**
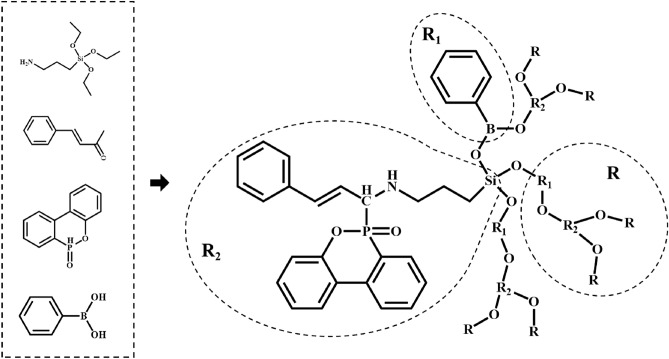
The synthesis route of DOPO-KhCPA.

In the second step, PA (12.2 g, 0.1 mol) and dichloroethane (134 ml) were added to a flask and stirred under nitrogen atmosphere until dissolved, followed by dropwise addition of precursor flame retardant (55.15 g, 0.1 mol) and dichloroethane (300 ml) to the above solution. The mixture was reacted at 80 ℃ for 8 h, then cooling to room temperature, the solution was filtered and concentrated on a rotary evaporator. After drying under vacuum at 50 °C for 12 h. DOPO-KhCPA was finally obtained with pulverized to give a yellow powder in 92% yield. The synthesis route of DOPO-KhCPA was shown in Fig. 1.

### Preparation of EP samples

Add the desired weight of EP to the reaction vessel and set the temperature of the frying pan to 120 °C. Heat and stir the EP until it becomes fluid, DOPO-KhCPA was added and continued to be stirred to dissolve it in the EP. After that, the temperature was adjusted to bring the mixture down to 75 °C and the curing agent (DDM) was added, after the curing agent had been dissolved the samples were placed in the vacuum oven to allow the bubbles to be removed. Finally, the mixtures were poured into molds of different sizes and treated with curing procedures (120 °C/2 h, 150 °C/2 h, 180 °C/2 h) to obtain EP samples that met the specifications of the test standards. The composition and curing procedures of EP composites was shown in Table 1.

**Table 1:** Composition of EP composites.

Samples	E-51 (g)	DDM (g)	DOPO-KhCPA (g)
Pure EP	80	20	0
DOPO-KhCPA-1	79.2	19.8	1
DOPO-KhCPA-3	77.6	19.4	3
DOPO-KhCPA-5	76	19	5

### Characterization

To analyze the chemical structure of the flame retardant, FTIR spectra of the reactants and products were collected using a Perkin-Elmer Spectrum Two instrument. In order to analyze the curing behavior of EP blends containing flame retardants and curing agents, the EP prepolymers were analyzed using the Perkin-Elmer DSC 4000 instrument. The experiments were conducted under an N_2_ atmosphere at a heating rate of 10 °C/min. To evaluate the thermal stability, thermogravimetric analysis (TGA) was performed using a Rigaku TG-DTA 8122. The samples were examined at a rate of 10 °C/min under a flow of N_2_ from 30 to 800 °C. According to ASTM D2863, JF-3 LOI tester (JiangNing Instrument, China) was used to measure the limiting oxygen index (LOI) value, the dimensions sheet were 120 mm × 10 mm × 4 mm. According to ASTM D3801, vertical burning testing for UL-94 ratings was performed using a CZF-3 combustion tester (JiangNing Instrument, China) with a specimen size of 125 mm × 13 mm × 3 mm. The combustion behavior of EP was measured at a heat flux of 50 kW/m^2^ using a cone calorimeter (Modisco Combustion Technology Instruments Co., Ltd.) according to ISO 5660-1 with a sample size of 100 mm × 100 mm × 3 mm. The dynamic mechanical thermal analyzer (DMA Q800, TA Instrument) was used to perform the dynamic mechanical analysis (DMA). The surface micromorphology of the residual carbon of EP materials after the CCT test was examined using scanning electron microscopy (SEM) on a Hitachi SU8000. The degree of graphitization of the residual carbon was analyzed using a LabRAM-HR Evolution Raman microscope with a 514.5 nm argon laser.

## Results and discussion

### Structural characterization

**Figure 2:**
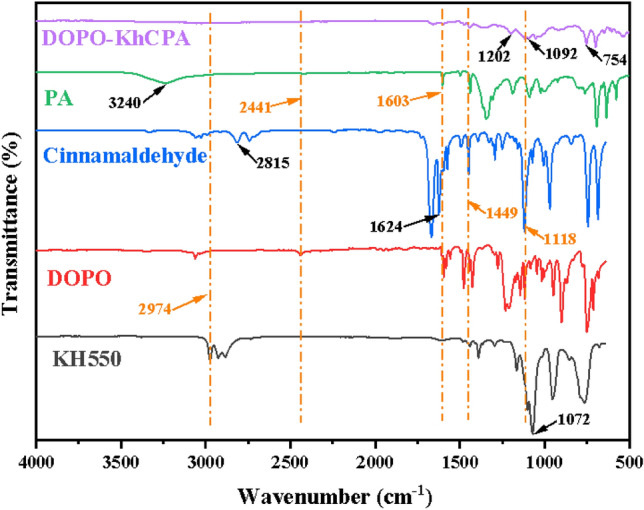
FTIR spectra of KH550, DOPO, PA, Cinnamaldehyde and DOPO-KhCPA.

The FITR was used to characterize the structure of DOPO-KhCPA, as shown in Fig. 2. The peaks in the FTIR spectrum of KH550 at 1072, 1624, and 2974 cm^−1^ belonged to Si–O–C, –NH_2_, and C–H, respectively^23^. The characteristic peaks of cinnamaldehyde at 1624 and 2815 cm^−1^ belonged to the C=C peak and –CHO peak, respectively^26^. In the spectrum of DOPO, the characteristic peak at 2440 cm^−1^ was attributed to the P–H bond, the P-Ph bond can be observed at 1118 cm^−1^, the P–O–Ph peak occurs at 900 cm^−1^, and the P = O peak appears at 1230 cm^−1^. Also, the –OH of phenylboronic acid can be seen at 3240 cm^−1^^27^. In the spectra of DOPO-KhCPA, the groups of P–O–Ph, P=O, P–Ph and P–C (754 cm^−1^), C–N and N–H were still observed. Moreover, the corresponding characteristic peaks of –OH, –NH_2_, C–O, and P–H groups involved in the reaction disappeared. These results demonstrated that DOPO-KhCPA was successfully synthesized.

### Curing behavior

**Figure 3:**
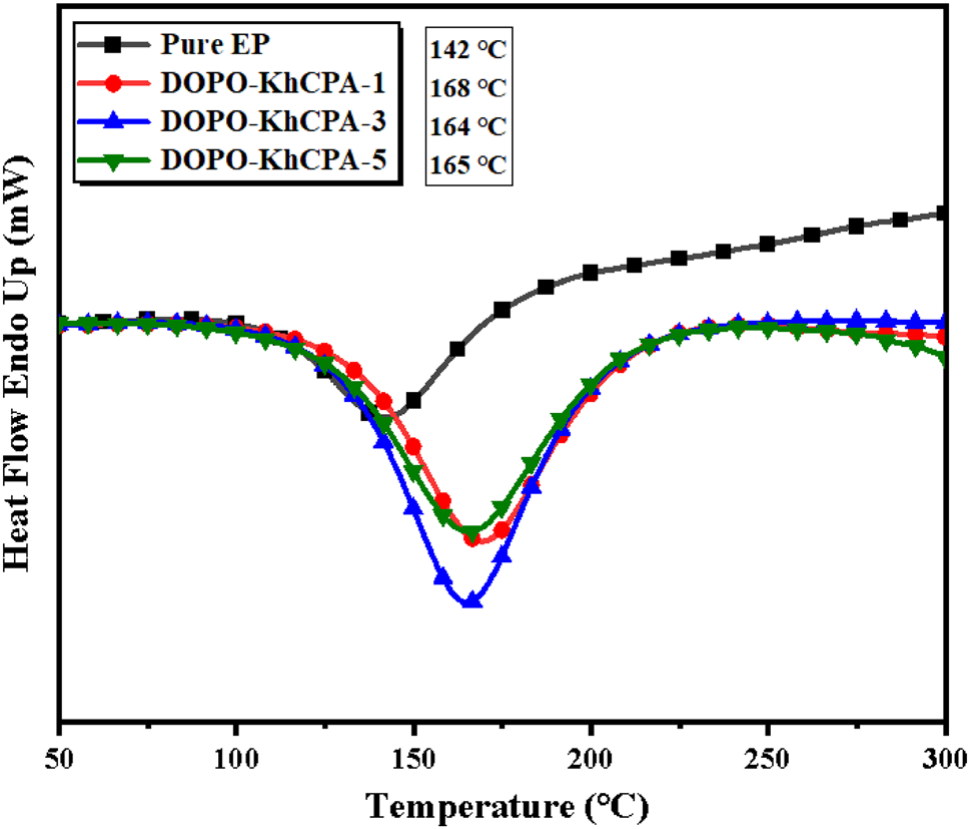
DSC curves of pure EP and DOPO-KhCPA composites.

The curing behavior of the EP prepolymer was analyzed using a differential scanning calorimeter (DSC). As shown in Fig. 3, the pure EP and the composites with the inclusion of the flame retardant displayed a downward peak, indicating the curing reaction process of the materials. It is important to mention that the curing peaks of the EP-cured compounds when DOPO-KhCPA was added, shift towards higher temperatures^26^. This indicated that DOPO-KhCPA hinders the curing process of the EP. This was due to the long chain structure between siloxane and phenylboronic acid in DOPO-KhCPA, which has strong stability and hinders the ring-opening reaction of EP, and the cross-linking groups form macromolecular chain structures, thus inhibiting the curing reaction of EP. In general, a more pronounced curing peak corresponds to a more vigorous exothermic reaction, which is undesirable for resin processing. The addition of DOPO-KhCPA to the curing system resulted in a noticeable broadening of the exothermic peak, in comparison to the curing peak of pure EP. This broadening of the exothermic peak helps to mitigate the concentrated exothermic reaction that occurs during the curing of EP, hence benifit to the actual processing program.

### Thermal stability

**Figure 4:**
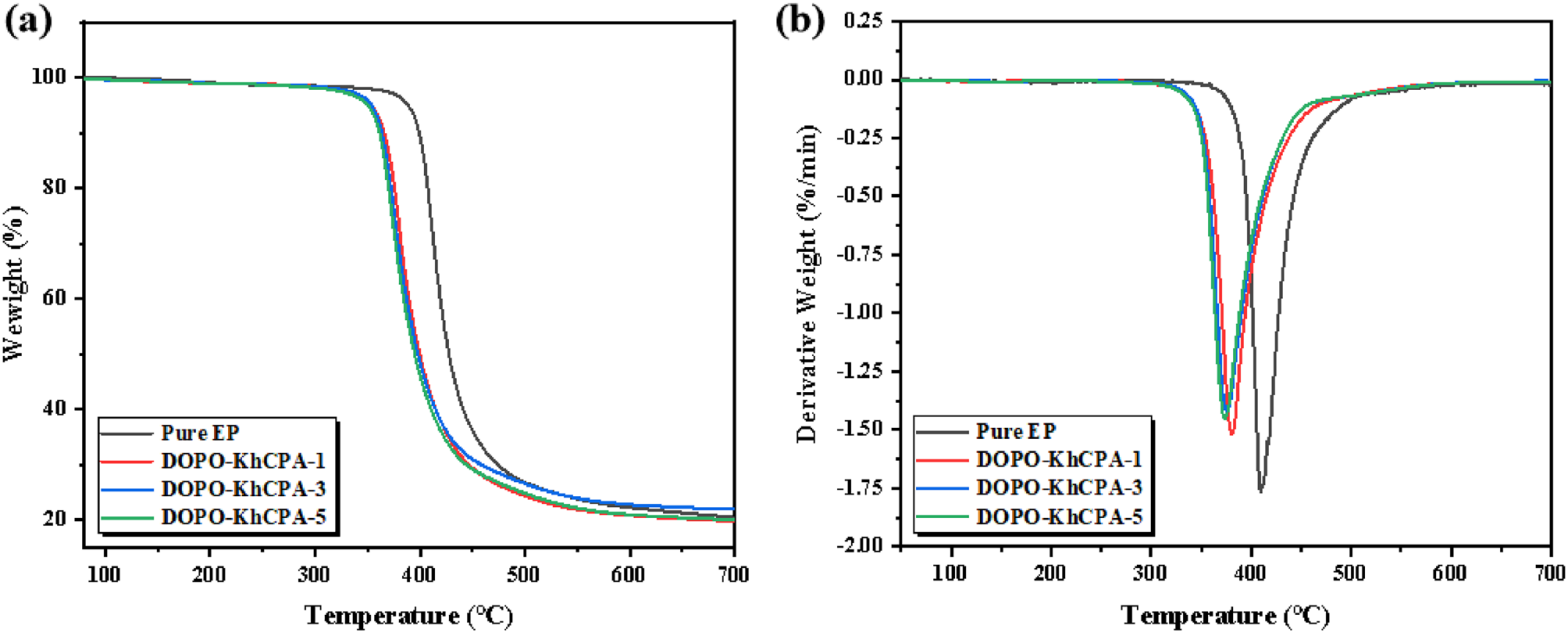
(**a**) TG and (**b**) DTG curves of pure EP and DOPO-KhCPA composites.

Thermal stability of EP composites was analyzed using thermogravimetric analysis (TG), and the curves and characteristic parameters were shown in Fig. 4 and Table 2, which mainly included temperature at which heat loss is 5% (T_5%_), maximum rate of weight loss (V_max_), the maximum weightlessness temperature (T_max_), and the residual carbon rate at 700 °C (C_y700_). Figure 4a demonstrated that the thermal decomposition stages of the EP contained with DOPO-KhCPA additives were all accelerated. This may be due to the phosphorus can catalyze the decomposition of EP in the early stages to form a carbon layer on the surface as quickly as possible that can be insulated from oxygen. Consequently, the flame retardant promotes the decomposition of the substrate into coke through catalytic action. The flame retardancy of DOPO-KhCPA was not affected, the residual char rate of the EP composites increased by 9.5% when 5% flame retardant was added, compared to the pure EP. The reason due to the catalytic matrix char building process of compounds containing phosphorus, and the presence of silicon can also facilitate the creation of a compact and enduring char layer. DOPO-KhCPA-1 and DOPO-KhCPA-3 composites have lower flame retardant additions, resulting in carbon residuals similar to pure EP. Figure 4b showed that the addition of flame retardant decreased the T_max_ of EP composites, this again validated the early protective effect of phosphorus^23^. Consequently, the dense carbon on the surface of the materials effectively slowing down the thermal decomposition process of the EP.

**Table 2:** TGA data of EP composites.

Sample	T_5%_ (℃)	T_max_ (℃)	V_max_ (%/min)	C_y700_ (%)
Pure EP	388	410	− 1.77	19.98
DOPO-KhCPA-1	350	379	− 1.51	19.98
DOPO-KhCPA-3	350	376	− 1.42	19.73
DOPO-KhCPA-5	350	373	− 1.44	21.88

### Flame retardant property

The flammability of EP encountering small flames was first tested using the LOI and vertical burning test, and the results of these tests are presented in Table 3. The pure EP exhibited inherent flammability, as seen by its low LOI value of 25.6%. Additionally, it failed to attain any flame retardant rating in the UL-94 test, as the samples continued to burn after ignition, accompanied by the occurrence of dripping. The flame retardant properties of the EP samples were significantly enhanced with the addition of DOPO-KhCPA. However, the LOI of DOPO-KhCPA composites with 1 wt% of DOPO-KhCPA was only 28.8%, and the UL-94 test only achieved V-1 rating. Notably, when DOPO-KhCPA was added at 3 wt% and 5 wt%, the LOI values increased to 30.3% and 32.0%, respectively, and both reached UL-94 V-0 rating without dripping. The combustion duration was shortened to 5 s and 3 s, respectively, indicating that the flame-retardant EP composites were capable of quickly extinguishing themselves after being removed from the source of the fire. The results show that the addition of DOPO-KhCPA can make EP exhibit good flame retardant effect when encountering flames.

**Table 3:** Flame retardant test data of pure EP and DOPO-KhCPA composites.

Sample	LOI (%)	UL-94 burning test		
		t_1_ + t_2_ (s)	Drips	Rating
Pure EP	25.6	Lasting burning	Yes	No Rating
DOPO-KhCPA-1	28.8	35	NO	V-1
DOPO-KhCPA-3	30.3	5	NO	V-0
DOPO-KhCPA-5	32.0	3	NO	V-0

**Figure 5:**
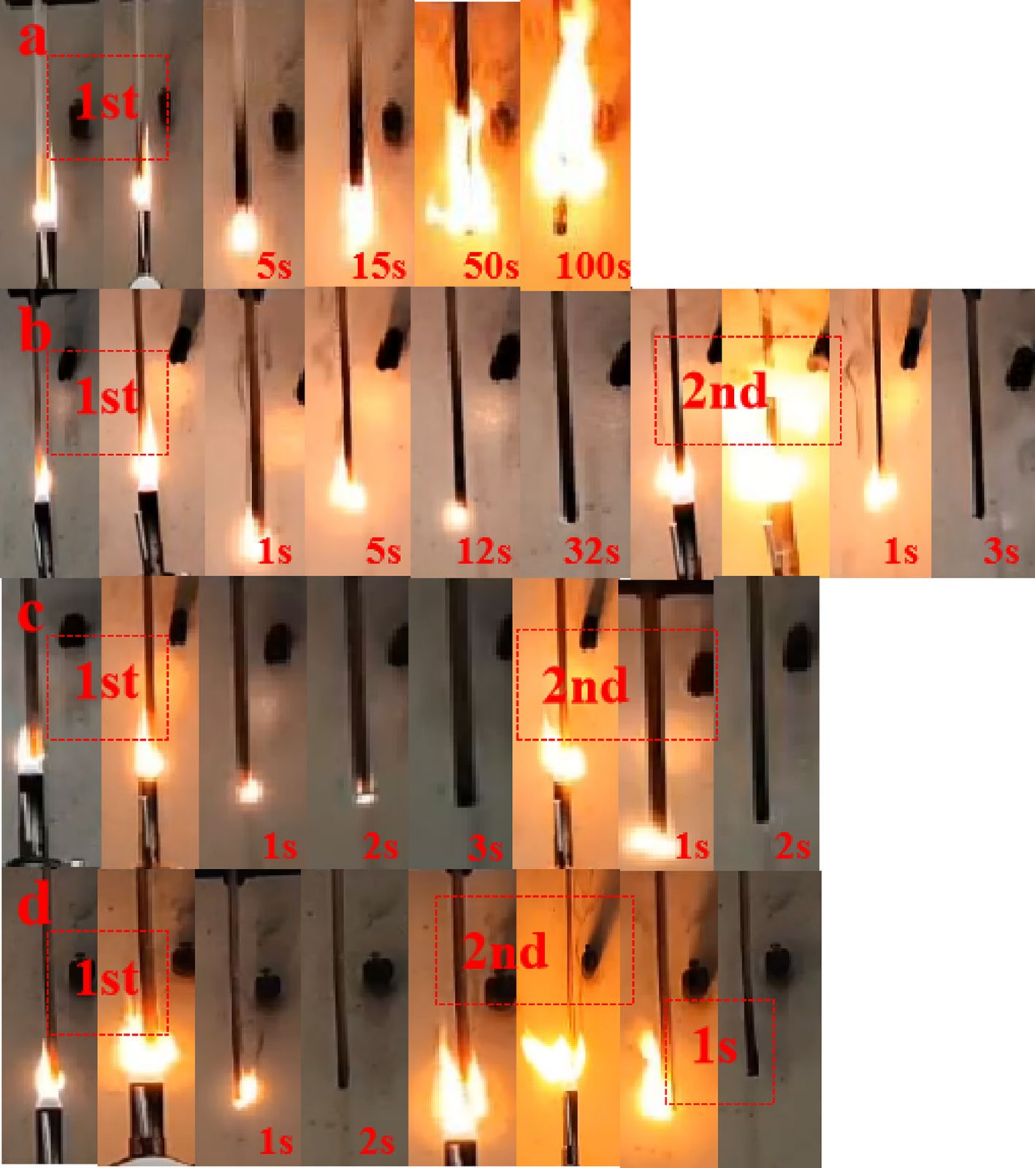
Digital photo of vertical burning test. (**a**) Pure EP; (**b**) DOPO-KhCPA-1; (**c**) DOPO-KhCPA-3; and (**d**) DOPO-KhCPA-5.

To examine the impact of DOPO-KhCPA on the fire resistance of EP, the vertical combustion test was documented using a digital camera. Figure 5 displays a video screenshot of the experiment, the graphic illustrates the quick combustion of pure EP upon ignition, accompanied by the occurrence of melt dripping. The introduction of DOPO-KhCPA has had a substantial impact on the burning characteristics of the EP. This is evident in the EP composites' ability to quickly self-extinguish after combustion, without any occurrence of dripping. In addition, it is also noted from Fig. 5 that EP concentrated gases from the combustion behavior of composite materials were continuously blown out from the interior of the samples, a phenomenon observed by Yang et al.^28^ in their study and named as the "blow-out effect". It was explained that the pyrolysis gases of the material were concentrated from the interior of the condensed layer, and the flame was extinguished by the blowing gases. The blow-out effect accelerates flame extinguishing and contributes to the flame retardancy of EP. In DOPO-KhCPA composites, the presence of phosphorus can play the gas phase flame retardant role to generate PO groups to capture free radicals, while the presence of boron and silicon can well form a stable carbon layer to achieve the role of protecting the matrix^29^.

**Figure 6:**
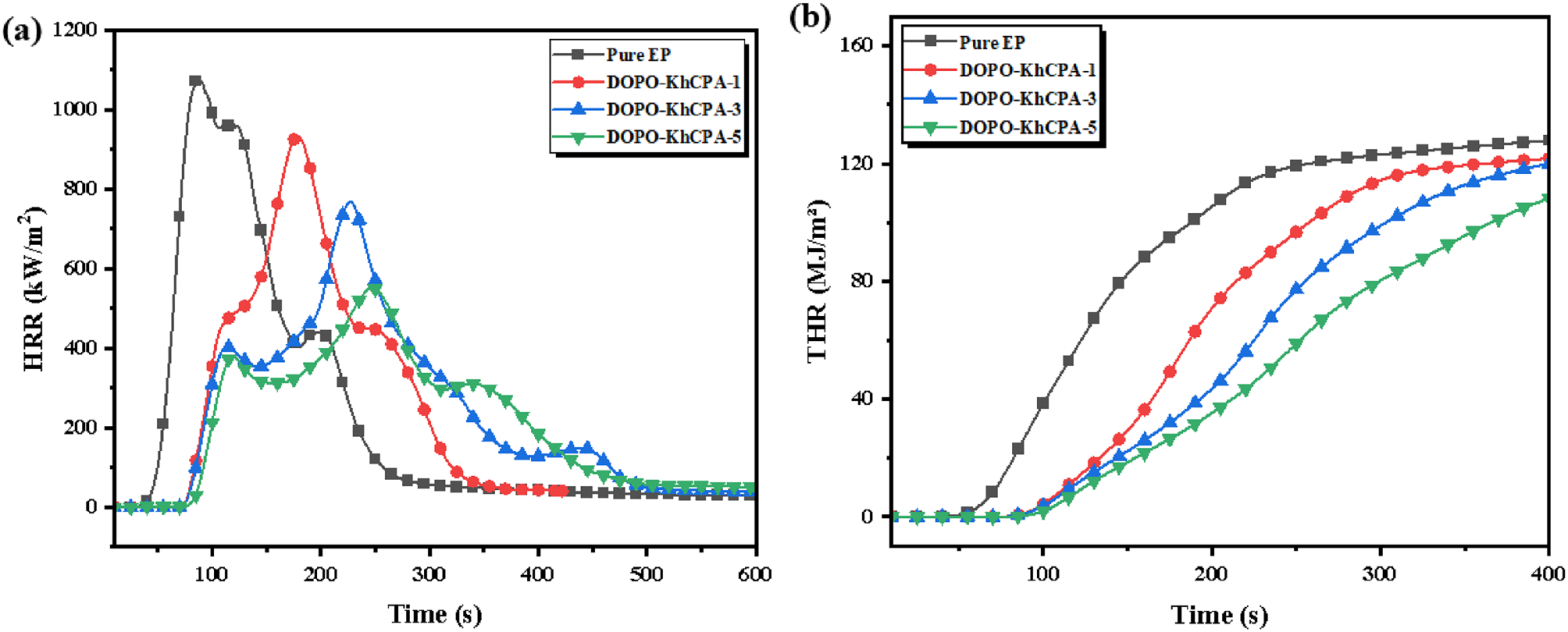
(**a**) HRR and (**b**) THR curves of pure EP and DOPO-KhCPA composites.

The Cone Calorimeter Test (CCT) is a highly effective experimental technique for investigating the combustibility of materials. The findings were presented in Table 4 and Fig. 6. Several relevant combustion parameters can be obtained from the CCT, including time to ignition (TTI), heat release rate (HRR), peak heat release rate (PHRR), total heat release (THR), and average effective heat of combustion (av-EHC). The length of the time to ignition (TTI) represents how well a material will flame retard when exposed to intense radiant heat. The less combustible material that evaporates, the longer the TTI will be. It can be seen that the TTI of EP composites gradually lengthens with increasing flame retardant content. According to the previous analysis of the TG data, this was attributed to the fact that DOPO-KhCPA was easily decomposed at lower temperatures, and in the decomposition process, it was mainly used to catalyze the condensed-forming process of the EP and to protect the gradual formation of the carbon layer, and thus the TTI was delayed. As shown in Fig. 6, the pure EP burns quickly after ignition. At about 100 s, its HRR started to increase sharply and its PHRR reached 1110 kW/m^2^, while the PHRR of the cured material with 1 wt% DOPO-KhCPA was reduced to 996 kW/m^2^. Continuing the addition of DOPO-KhCPA, the PHRR of the EP cured material was further reduced, and the PHRR of DOPO-KhCPA-5 composite was reduced to 569 kW/m^2^, representing a reduction of approximately 48.7% compared to pure EP. Similarly, the THR of various samples likewise exhibited a considerable decrease when the amount of DOPO-KhCPA added increased. The THR of pure EP attained a value of 127 MJ/m^2^. The heat release of the EP material was significantly suppressed by DOPO-KhCPA, as evidenced by a 7.2% decrease in the THR of DOPO-KhCPA-5 composites compared to pure EP. The av-EHC value reflects the relationship between exotherm and mass loss, and is an important indicator for evaluating the flame retardant effect of flame retardant products in the gas phase. It can be seen that the av-EHC values showed almost the same trend with the THR values. This suggests that the excellent flame retardancy of EP composites stems from the role played by flame retardants in the gas phase. It dilutes oxygen and those flammable gases by generating non-flammable gases, and the more compact residual carbon due to the cross-linked interpenetrating network structure will further reduce the leakage of flammable and non-flammable gases. The char layer inhibited the combustion of the EP, effectively preventing the transfer of heat to the interior of the resin, further inhibiting the pyrolysis of the EP and reducing the burning intensity^30–32^.

**Table 4:** CCT parameters for pure EP and DOPO-KhCPA composites.

Sample	TTI (s)	PHRR (kW/m^2^)	THR (MJ/m^2^)	av-EHC MJ/kg)
Pure EP	38	1110	127	26
DOPO-KhCPA-1	47	996	121	24
DOPO-KhCPA-3	44	869	135	23
DOPO-KhCPA-5	40	569	117	22

### Dynamic thermodynamic property

**Figure 7:**
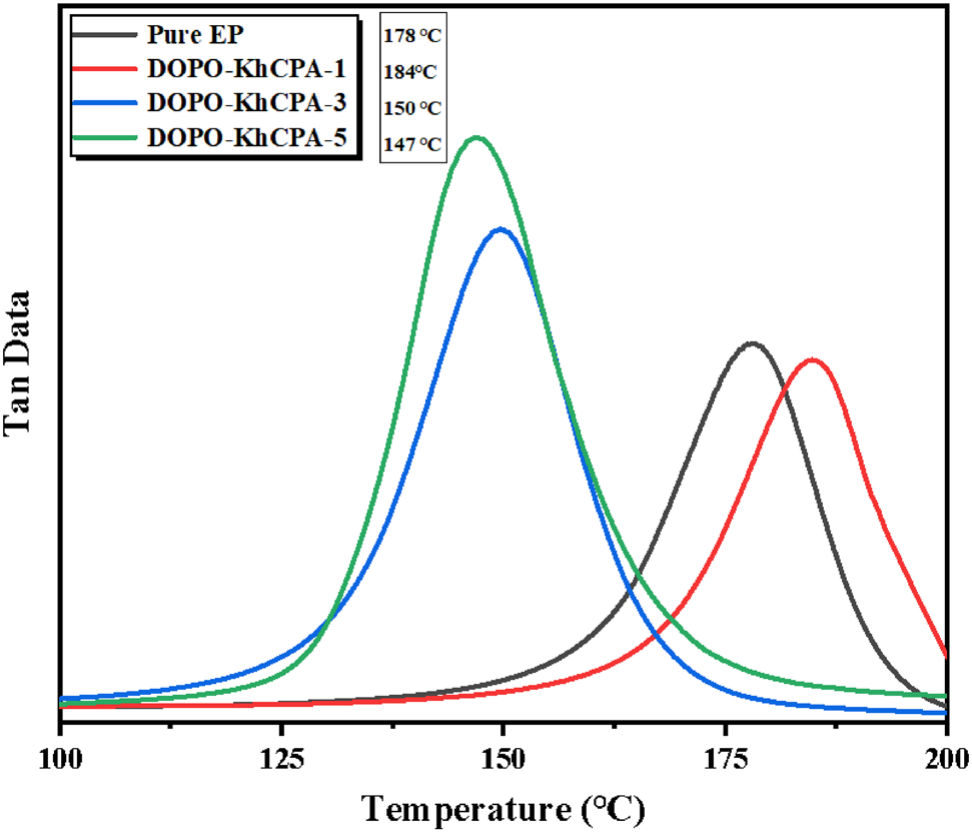
DMA curves of pure EP and DOPO-KhCPA composites.

The dynamic mechanical properties of the EP composites were assessed using DMA, as depicted in Fig. 7. The peaks observed in the curves correspond to the glass transition temperature (Tg), which exhibited a strong correlation with the rigidity of the EP network's backbone, the density of cross-linking, and the interplay between the filler and the matrix^33^. The Tg results showed that the Tg of DOPO-KhCPA-1 was increased compared with that of the pure EP, which was mainly attributed to the double-bonded structure, the aromatic heterocycles, and other stiffening groups in DOPO-KhCPA that would have a certain constraining effect on the main chain of the EP. As the content of DOPO-KhCPA increased, the Tg of DOPO-KhCPA-3 and DOPO-KhCPA-5 decreased, which was mainly attributed to the content of DOPO-KhCPA continues to rise. The incorporation of excessive small molecule flame retardants would have a certain negative impact on the cross-linking density of the EP. The decrease in the crosslink density of EP resulted in a significant decrease in the Tg of EP. Simultaneously, the Si–O–B structure contained in the flame retardant itself was extremely flexible, which would lead to the decrease in the rigidity of the flame retardant itself. Overall, low addition of DOPO-KhCPA contributes to the dynamic mechanical properties of EP.

### Flame retardant mechanism analysis

**Figure 8:**
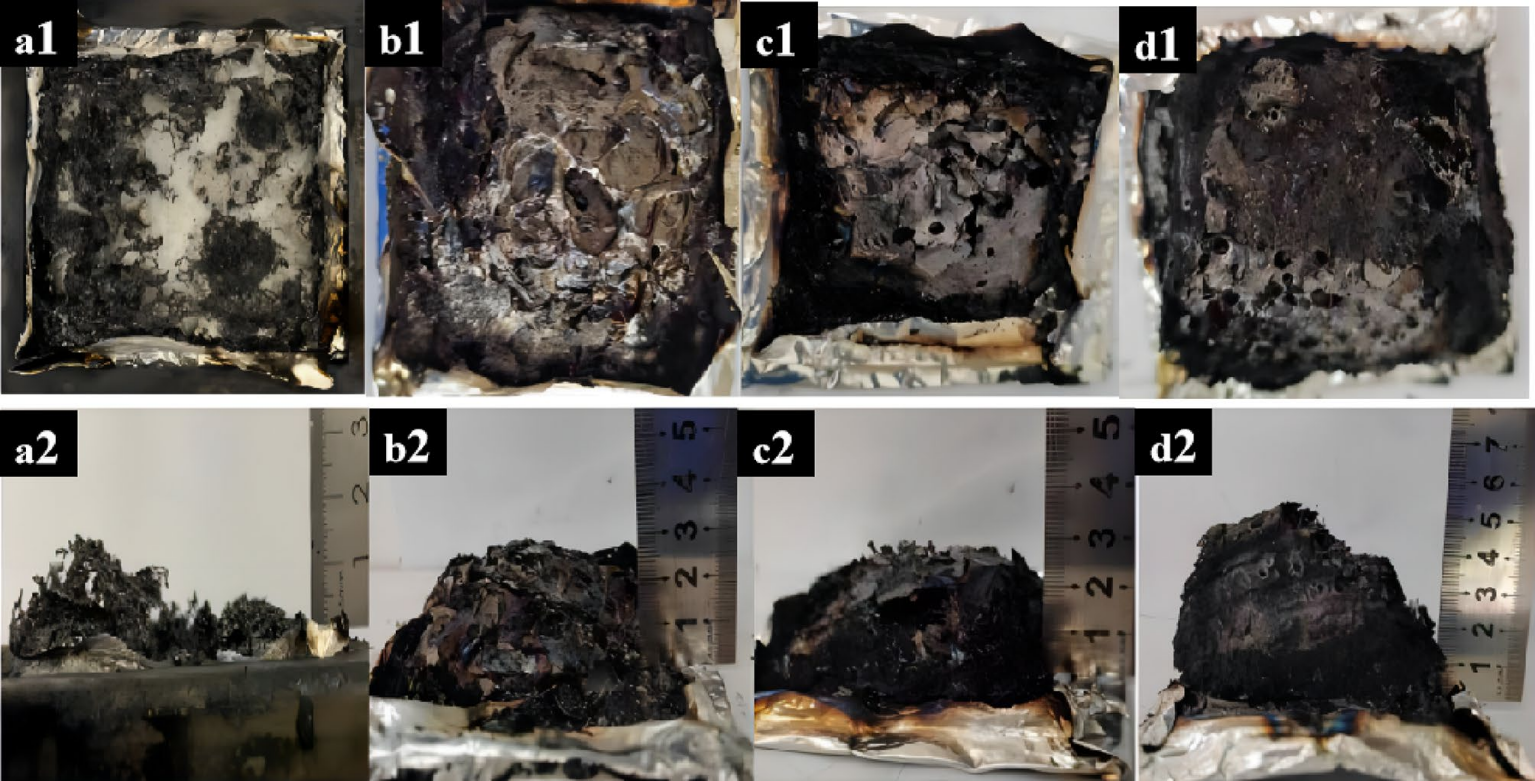
Electronic digital photographs after CCT. (**a**_**1**_–**a**_**2**_) Pure EP, (**b**_**1**_–**b**_**2**_) DOPO-KhCPA-1, (**c**_**1**_–**c**_**2**_) DOPO-KhCPA-3, and (**d**_**1**_–**d**_**2**_) DOPO-KhCPA-5.

Characterization of the carbonaceous material remaining after combustion of EP composites gives the flame retardant mechanism in the condensed phase. Figure 8 displays digital photos depicting the remaining char of the EP composites following CCT. It is evident that a minimal quantity of residual carbon is left after the complete combustion of pure EP, and the tin foil at the bottom was completely burned. The amount of carbon remaining after the burning of the EP composites showed a substantial rise as the DOPO-KhCPA addition increased. This suggests that DOPO-KhCPA possesses exceptional catalytic properties for carbon production. And the residual carbon of DOPO-KhCPA-5 composites exhibited an expanded honeycomb carbon layer. The dense carbon layer acts as a barrier, effectively isolating external combustion-fueled heat and oxygen. This was due to the fact that some small molecule gases generated during combustion accumulate in the char layer, and the increasing accumulation of gases leads to the expansion of the char layer. When it reached the capacity limit of the char layer, the gases break through the char layer and blow out to the outside, resulting in the "blowing out effect". In addition, the presence of boron and silicon of the DOPO-KhCPA enhanced the char formation ability of the EP, which helps to facilitate the effect of the flame retardant^34,35^.

**Figure 9:**
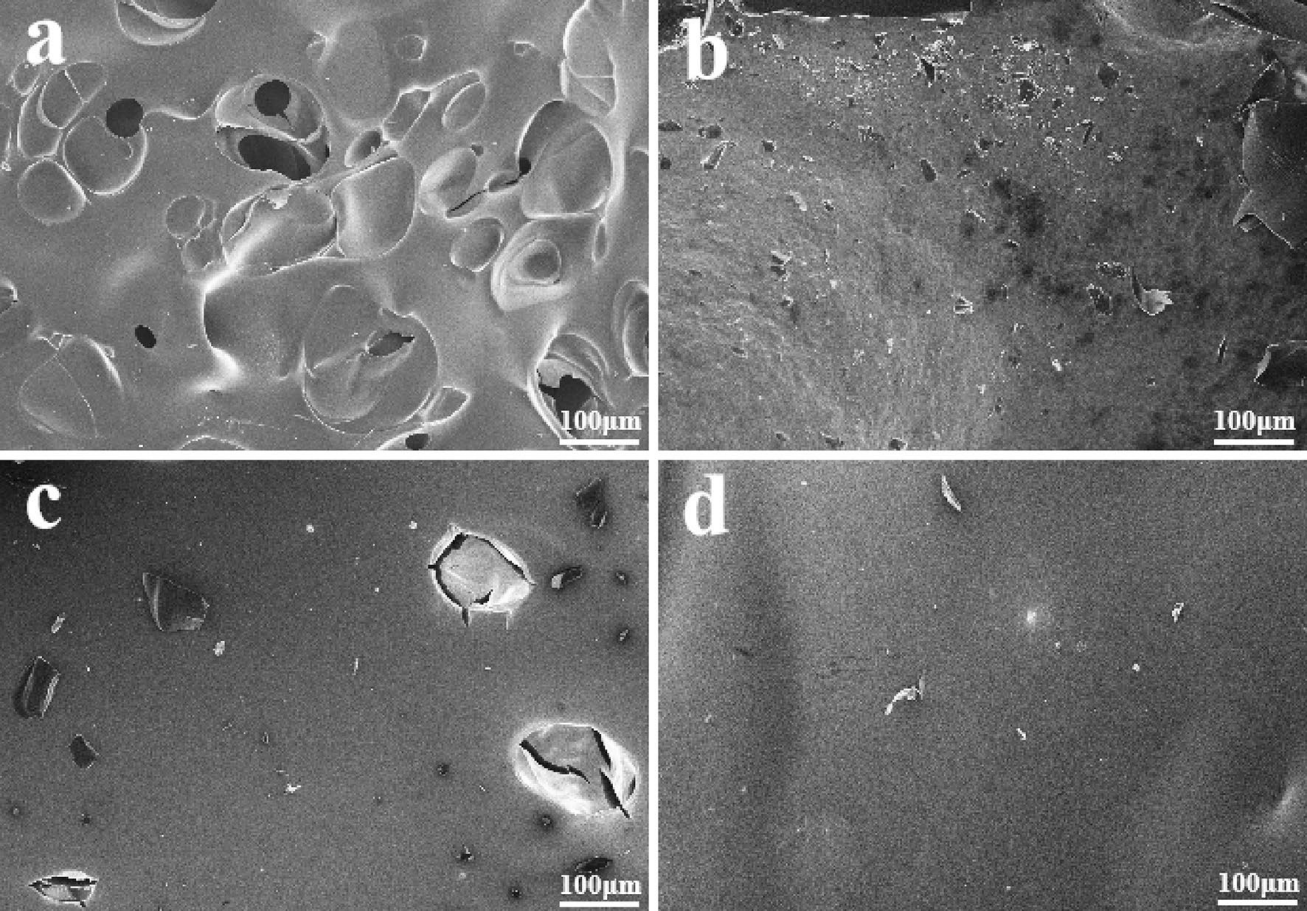
SEM photo of residual carbon. (**a**) Pure EP, (**b**) DOPO-KhCPA-1, (**c**) DOPO-KhCPA-3, and (**d**) DOPO-KhCPA-5.

In addition, the residual carbon of the EP was analyzed morphologically using a SEM to examine the microstructure of the remaining carbon after the CCT. Figure 9 demonstrates that the residual carbon in pure EP exhibits numerous cracks and pores, making it challenging to hinder the transport of oxygen and heat during combustion due to this fragmented carbon layer. The DOPO-KhCPA composites exhibited a char layer with a very intact structure, featuring small cracks and pores, which became flatter as the amount of DOPO-KhCPA added increased. This is due to the catalytic effect of elemental phosphorus, which enhances the ability to produce carbon and has an inhibitory effect on the combustion reaction. The presence of boron and silicon components in the DOPO-KhCPA structure also enhancing the development of the carbon layer during combustion. In addition, DOPO-KhCPA has a double-bond structure, which can spontaneously form a large-scale chain structure. This structure can be interspersed with EP to form an interpenetrating crosslinked network, which effectively strengthens the residual carbon. Figure 9d showed that the carbon layer of DOPO-KhCPA-5 displays a uniform structure without any obvious gaps or breaks. This smooth carbon layer acts as a shield to protect the underlying DOPO-KhCPA from thermal radiation, thus improving its refractoriness.

**Figure 10:**
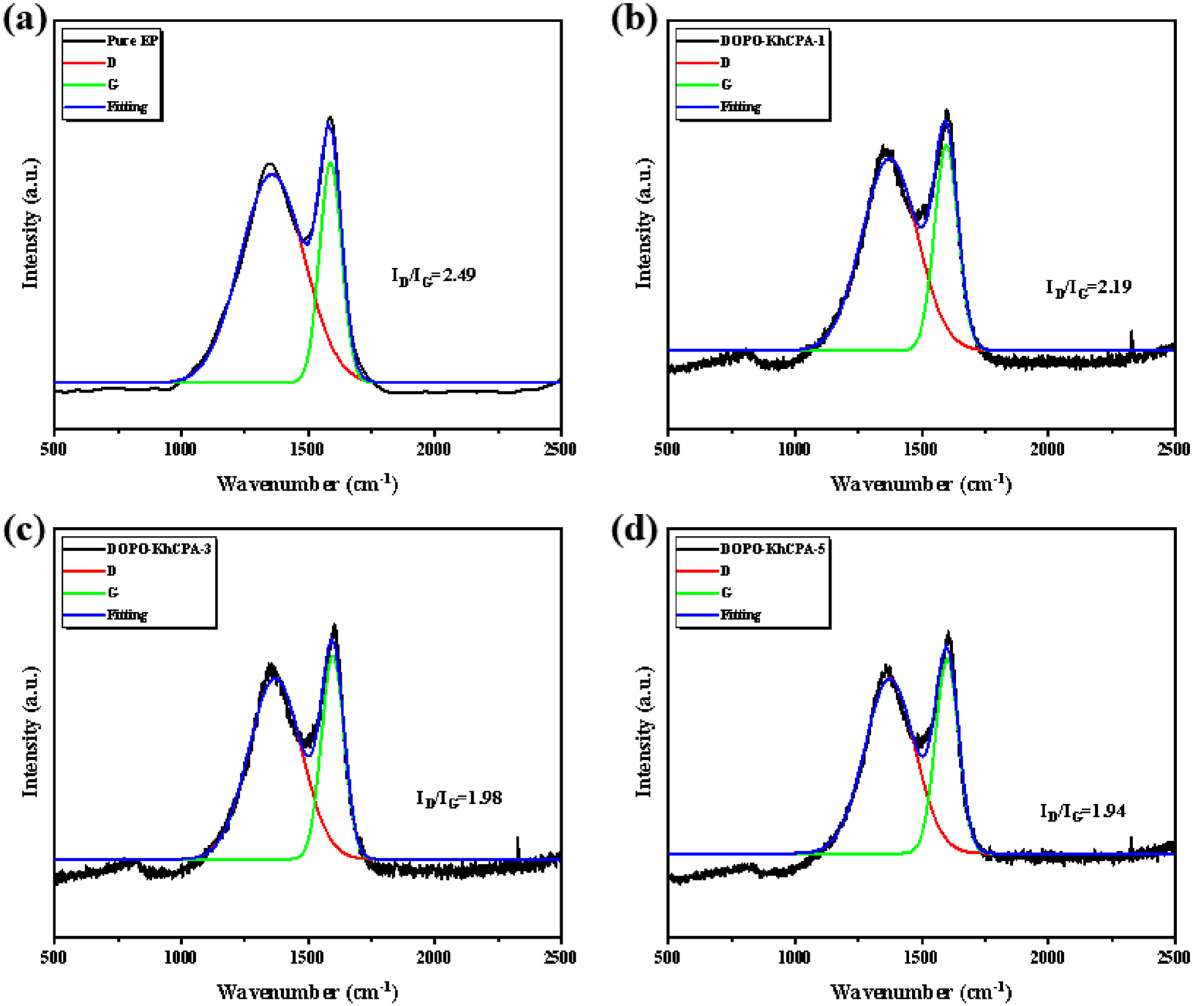
Raman spectra of residual carbon. (**a**) Pure EP, (**b**) DOPO-KhCPA-1, (**c**) DOPO-KhCPA-3, and (**d**) DOPO-KhCPA-5.

Furthermore, to acquire the hardness data of the residual carbon, we conducted Raman spectroscopy. Figure 10 illustrates the Raman analysis conducted on the residual carbon of both pure EP and DOPO-KhCPA composites. The degree of graphitization of the carbon material can be quantified as the intensity ratio of the two bands (I_D_/I_G_)^36,37^. The smaller the value, the more dense and thermally stable the carbon layer is^38,39^. Figure 10 showed that the I_D_/I_G_ value decreased as the amount of DOPO-KhCPA was increased, the lowest I_D_/I_G_ value is observed for DOPO-KhCPA-5 composites. As the flame retardant content increased, the carbon layer produced by the material after combustion was more thoroughly graphitized, which made the residual carbon denser and enhances the thermal stability of EP materials. The Raman results suggested that DOPO-KhCPA enhanced the completeness of the char layer, while also insulating heat transfer. Consequently, DOPO-KhCPA exhibits excellent protective effect in the condensed phase.

**Figure 11:**
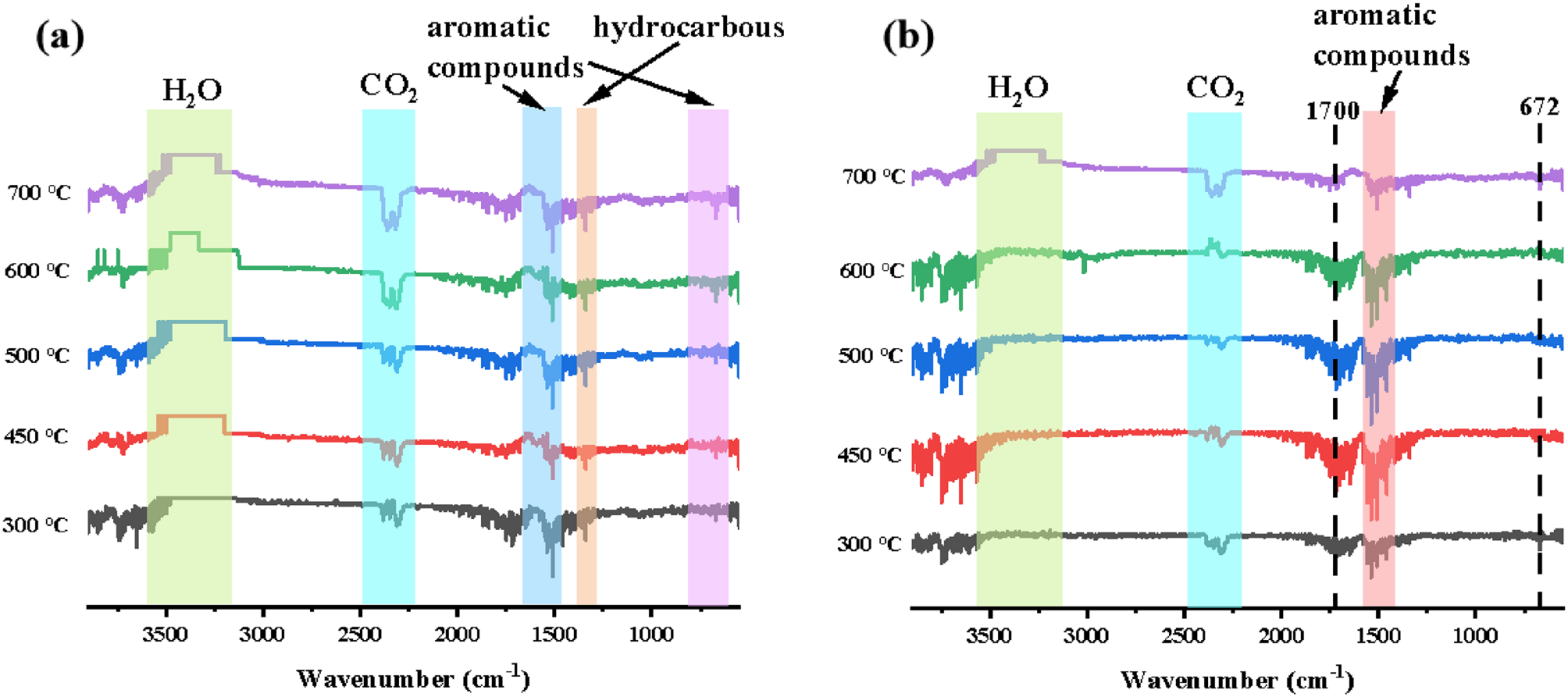
FTIR spectra of pyrolysis products of (**a**) pure EP and (**b**) DOPO-KhCPA-5 composites at different stages of decomposition.

Further, thermogravimetric infrared analysis was used to analyze the gas-phase flame retardant mechanism of the flame retardant, which is embodied in the pyrolysis products of the EP composites at elevated temperatures. Figure 11 displayed the FTIR spectra of pure EP and DOPO-KhCPA-5 composite at various temperatures^23^. The primary degradation products of the pure EP consisted of hydrocarbons (1100–1300 cm^−1^ and 2800–3200 cm^−1^), CO_2_ (2359 cm^−1^), compounds containing hydroxyl groups (3500–3700 cm^−1^), and aromatic compounds (749 cm^−1^, 830 cm^−1^, 1508 cm^−1^, and 1598 cm^−1^). The spectra of DOPO-KhCPA-5 showed similar peaks compared to the spectra of the pure EP. However, a significant change in the ratio between the peaks was observed. It is noteworthy that the peaks of CO_2_ were reduced, which indirectly proved that DOPO-KhCPA can effectively reduce the amount of CO_2_ was released and achieve the purpose of reducing the flue gas content. The increase of the peak at 672 cm^−1^ is attributed to the presence of the DOPO group. Furthermore, Phosphorus-containing flame retardants form products containing P–O–Ph bonds during decomposition, which help to dilute flammable gases and capture flammable free radicals to retard the expansion of the reaction, thereby effectively protecting the EP matrix from sustained combustion.

### Mechanical property

**Figure 12:**
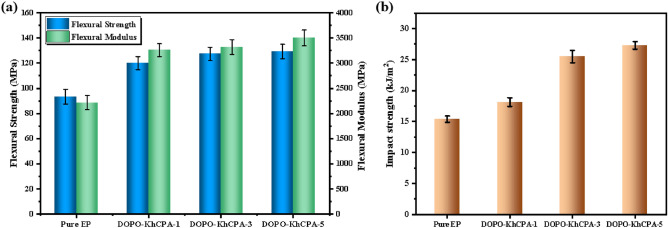
Tensile strength and tensile modulus of pure EP and DOPO-KhCPA composites.

To assess the compatibility of DOPO-KhCPA flame retardant with EP composites, we examined the mechanical properties of DOPO-KhCPA composites and presented the results of the tensile and impact test in Fig. 12. The incorporation of flame retardants in EP composites resulted in enhanced mechanical characteristics. The impact strength of EP composites gradually increases with increasing flame retardant content. The tensile strengths of DOPO-KhCPA-1 and DOPO-KhCPA-3 composites were seen to rise by 28.7% and 36.6%, respectively, compared to pure EP. Furthermore, the DOPO-KhCPA-5 composite exhibited the maximum tensile strength (129 MPa), indicating that the DOPO-KhCPA flame retardant is effectively dispersed throughout the EP matrix. The cross-linkable groups in DOPO-KhCPA will interpenetrate and cross-link with EP, thus increasing the entanglement of the material. Additionally, the presence of silicon elements allows for the formation of a stable Si–O–Si structure^27^, which keeps the mechanical properties of the EP matrix. In general, the inclusion of DOPO-KhCPA has a positive impact on the mechanical characteristics of the EP composites.

## Conclusion

In this study, a novel multi-element synergistic flame retardant was developed to providing efficient flame retardancy while mitigate the adverse effects of flame retardants on the mechanical properties of substrates. For this purpose, aldehydes with double-bond structures were used for grafting reactions with elements such as phosphorus, nitrogen and boron. A multi-element synergistic flame retardant named DOPO-KhCPA was synthesized, which has both flexible and macromolecular chains. The addition of 5 wt% of the flame retardant resulted in the most significant improvement in the flame retardant and flexural properties of EP. Compared with pure EP, PHRR and THR of DOPO-KhCPA-5 were reduced by 48.7% and 7.2%, respectively, and flexural strength increased to 129 MPa. The flame retardant mechanism of DOPO-KhCPA involves the catalytic role of phosphorus in promoting carbon formation in the EP matrix. Nitrogen can be gas phase flame retardant by thermally decomposing to produce a non-combustible gas to dilute the combustion environment. In addition, the combination of the flame-retardant elements silicon and boron promotes the densification and stabilization of the protective carbon layer, which acts as a barrier to prevent combustion-enhancing oxygen and heat from entering the combustion interior. The double-bond in the flame retardant formed a dense network inside the EP through cross-linking, which effectively reduced the impact of the addition of flame retardant on the mechanical properties of EP.
